# DCVax-L Vaccination in Patients with Glioblastoma: Real Promise or Negative Trial? The Debate Is Open

**DOI:** 10.3390/cancers15123251

**Published:** 2023-06-20

**Authors:** Lidia Gatto, Vincenzo Di Nunno, Alicia Tosoni, Stefania Bartolini, Lucia Ranieri, Enrico Franceschi

**Affiliations:** 1 Department of Oncology, Azienda Unità Sanitaria Locale (AUSL) Bologna, 40139 Bologna, Italy; lidia.gatto83@gmail.com (L.G.); vincenzo.dinunno@ausl.bologna.it (V.D.N.); 2 Nervous System Medical Oncology Department, IRCCS Istituto delle Scienze Neurologiche di Bologna, 40139 Bologna, Italy; a.tosoni@isnb.it (A.T.); stefania.bartolini@isnb.it (S.B.); lucia.ranieri@ausl.bologna.it (L.R.)

**Keywords:** DCVax-L, dendritic cell vaccination, glioblastoma, immuotherapy

## Abstract

**Simple Summary:**

Glioblastoma is the most common of primary brain tumors, accounting for approximately 50% of intracranial malignancies. It is an aggressive neoplasm with a poor prognosis. To date, the standard of care is a treatment involving maximal surgery, radiotherapy concurrent with and followed by maintenance chemotherapy with temozolomide. Despite this multimodal approach and the continuous advances in molecular biology, median survival is 13–14 months and 5-year survival does not exceed 10% of patients. Therefore, the need to develop new treatments that can impact the survival of glioblastoma patients is urgent. After decades of research failures, immunotherapy timidly begins to give the first results in the treatment of this tumor. The publication of the phase III study on the use of the dendritic cell vaccine DCVax-L in glioblastoma has aroused much interest in neuro-oncology. We report the promising results of this trial, which, however, is worthy of a critical debate regarding both the special study design and the authors’ conclusions.

**Abstract:**

The lack of significant improvement in the prognosis of patients with GB over the last decades highlights the need for innovative treatments aimed at fighting this malignancy and increasing survival outcomes. The results of the phase III clinical trial of DCVax-L (autologous tumor lysate-loaded dendritic cell vaccination), which has been shown to increase both median survival and long-term survival in newly diagnosed and relapsed glioblastoma, have been enthusiastically received by the scientific community. However, this study deserves some reflections regarding methodological issues related to the primary endpoint change, the long accrual period, and the suboptimal validity of the external control population used as the comparison arm.

## 1. Introduction

Glioblastoma (GB) is the most aggressive CNS tumor, with an incidence rate of about 8 per 100,000 people and an average survival of approximately 13–14 months [1]. The 5-year survival rate is less than 5% [2]. GB recurrence is near-universal despite surgical removal and multimodal treatments [3]. Although many trials have been concluded in the last decades, almost all therapeutic agents have proved ineffective in increasing survival and maximal surgical resection, followed by concomitant radio-chemotherapy and adjuvant temozolomide, remains the established therapeutic standard of care [4]. With the introduction of loco-regional tumor-treating fields (TTFields) therapy, an innovative strategy consisting of low-intensity alternating electric fields approved for the treatment of recurrent and newly diagnosed GB, the hope to increase PFS and OS while improving quality of life, fired up [5,6].

Immunotherapy has revolutionized the outcome of various solid tumors [7,8,9,10,11,12], but it has proven ineffective in the treatment of GB [13,14,15]. GB is an extremely heterogeneous cancer and poses several difficult challenges to the success of immunotherapy. First of all, it is an immunologically “cold” tumor, characterized by a tumor microenvironment (TME) enriched with immunosuppressive cytokines including transforming growth factor-beta (TGF-B), interleukine-6 (IL-6), IL-10, and immune regulatory cells (T-regulatory lymphocytes, ‘protumoral’ M2 macrophages, myeloid-derived suppressor cells, and tumor-associated macrophages) that turn off inflammation and disable an effective immune response by T CD8+ lymphocytes and natural killer (NK) cells [16,17,18,19,20,21]. Another crucial issue is the presence of both intra-tumor and inter-tumor heterogeneity in GB cells, resulting in the production of “poor quality” neoantigens, named “sub-clonal” neoantigens (present only in a subset of tumor cells and not in all tumor cells), that are much less immunogenic than clonal neoantigens and inadequate to elicit an effective immune response [22]. The secretion of immunosuppressive factors by glioma cells and the chronic exposure to “sub-threshold” antigenic stimulation lead T lymphocytes to a state of metabolic “exhaustion”, which renders them inactive [23,24]. Furthermore, GB cells overexpress the checkpoint protein PD-L1, which binds its ligand PD-1 in microglia, resulting in down-regulation of T-lymphocyte proliferation and cytotoxic activity [25]. The big bet of immunotherapy in neuro-oncology is controlling the enormous heterogeneity of GB and finding a way to modify the TME, “manipulating” it to obtain an effective antigen-specific immune response without triggering a severe intracranial local inflammatory reaction.

Dendritic cell vaccination is a type of immunotherapy consisting of autologous innate immune cells, dendritic cells, pulsed with autologous tumor lysate that has emerged as a promising novel treatment because it is the paradigm of tailored therapy, producing active immune products manufactured on the specific tumor antigens of each patient [26,27]. Promising results have been reported for sipuleucel-T [28], approved by the FDA in 2010 for metastatic castration-resistant prostate cancer (not in Europe due to the modest advantage offered compared to other therapies), and for other dendritic vaccines applied to various types of solid and haematologic malignancies [29,30,31,32]. To date, many dendritic cell vaccinations are in the experimental stage for melanoma (NCT00390338), breast cancer (NCT04348747), hepatocellular cancer (NCT04912765), and colorectal cancer (NCT03827967). Active immunotherapy with dendritic cell vaccination in GB has been observed since 1999, demonstrating reduced tumor growth, prolonged survival, and antigen-specific cytotoxic T-CD8+ lymphocyte responses in murine models and early-stage clinical trials [33,34,35,36,37].

Liau et al. [38,39] reported the results of a phase III clinical trial (NCT00045968) testing DCVax-L vaccine on patients with either newly diagnosed or recurrent GB to assess whether autologous tumor lysate-loaded dendritic cell vaccine administered in addition to standard of care (SOC) may improve survival outcomes.

DCVax-L is a highly personalized vaccination that uses tumor lysate as a source of antigens and uses the patient’s autologous dendritic cells harvested by leukapheresis and then expanded in vitro.

## 2. Study Design

The study was a prospective multicentric randomized double-blind phase III trial, involving 331 patients (232 patients in the DCVax-L arm and 99 patients randomized to the placebo group) among 94 centers in 4 countries (US, Canada, UK, and Germany) [39]. The trial began in 2007, was suspended from 2008 to 2011 for economic reasons, and ended in 2015. Approximately 90% of patients were randomized from 2012 to 2015. 

Patients with newly diagnosed GB were to be randomized 2:1 to standard radio-chemotherapy with either placebo or DCVax-L. Progression-free survival (PFS) was selected as the primary endpoint. The study design included the cross-over, so all patients could receive DCVax-L following tumor recurrence. With cross-over at progression, overall, 90% of patients from the two arms, experimental and control, received DCVax-L.

## 3. Results

A first report of the interim data of the ITT (intention to treat) population was published in 2018; investigators did not report the results for PFS, the primary endpoint, explaining that PFS could not be assessed for that publication and that it would be analyzed later to allow for central, multi-factorial assessment by an expert panel [39]. The authors only reported survival data, underscoring the high percentage of long-term survivors. In particular, the median OS (mOS) in the ITT population was 23.1 months (95% CI 21.2–25.4). mOS in the subgroup of patients with methylated O-6-methylguanine-DNA methyltransferase (MGMT) was 34.7 months (95% CI 27.0–40.7). The three-year survival percentage was 25.4%. In addition, a subpopulation of extended survivors (n = 100) with mOS of 40.5 months, only partially justified by favorable prognostic factors, was reported (in this subgroup, about 30% of patients were <50 years old, 70% underwent complete surgical resection, and 65% had MGMT-methylated tumors) [39].

Although it was a double-arm phase III trial, a single survival curve was presented, an anomalous fact for a randomized study.

In 2023, the final results of the study will be published [38]. Investigators stated that PFS was not an appropriate endpoint for two reasons: first, the placebo group was excessively depleted by the cross-over (overall, 90% of patients from the two arms received DCVax-L); second, the interpretation of PFS data was difficult due to the phenomenon of pseudoprogression. Therefore, the study design was changed and adapted at later stages of the research project, and the primary endpoint of the study was changed from PFS to OS. 

An external control group was created as a comparator group using several selected randomized clinical trials, both in the adjuvant and recurrence settings. 

A systematic literature review was conducted to identify relevant studies in newly diagnosed GB and recurrent GB, respectively, to provide comparator control populations. Five phase III studies [6,40,41,42,43], each with a control arm treated with the standard radiotherapy and temozolomide regimen, were selected as the comparator control group for the newly diagnosed setting (for a total of 1366 patients). As a consequence of the cross-over design, approximately 90% of patients were treated with DCVax-L thus, the two arms (DCVax-L and placebo) from the original study were merged together in the OS analyses. 

A separate survival analysis of patients receiving DCVax-L at progression (recurrent GB) was performed; for this purpose, ten phase III comparator studies (for a total of 640 patients treated with SOC, such as lomustine or bevacizumab) [44,45,46,47,48,49,50,51,52,53] were selected as the control group. Practically, they created a new study population (recurrent GB), on which they conducted analyses that were not initially planned.

Thus, they finally compared OS in patients with newly diagnosed GB and recurrent GB treated with DCVax-L plus SOC vs. external control patients treated with SOC. In order to minimize biases due to confounding factors in this adapted study design and imbalances in patients’ characteristics, the authors performed a matching-adjusted indirect comparison.

Therefore, this study is configured, after modification of the study design and of the primary endpoint, as a non-randomized single-arm trial with an external control group.

In this second report recently published, finally, PFS data were reported and were not encouraging since DCVax-L + SOC performed worse than SOC alone. The median PFS was 6.2 months for the DCVax-L arm and 7.6 months for the placebo group (*p* = 0.47). Nevertheless, significant OS improvement was registered in both newly diagnosed and relapsed GB patients treated with DCVax-L + SOC compared with the external control group that received SOC alone. 

Patients with newly diagnosed GB treated with DCVax-L + SOC survived 19.3 months (95% CI, 17.5–21.3) compared to 16.5 months (95% CI, 16.0–17.5) for the control group (*p* = 0.002). Patients with recurrent GB treated with the vaccine survived 13.2 (95% CI, 9.7–16.8) months versus 7.8 (95% CI, 7.2–8.2) months for the control group (*p* < 0.001). 

OS was improved in patients with newly diagnosed MGMT-methylated GBs receiving DCVax-L (30.2 months) compared with external control patients (21.3 months) (HR, 0.74; 98% CI, 0.55–1.00; *p* = 0.03).

Survival at 48 months was 15.7% in the experimental group vs. 9.9% in the control group, with the longest survivor still alive 8 years after randomization. Survival at 60 months was 13% in the investigational arm and 5.7% in the external control group.

The survival advantage of DCVax-L was better in poor prognosis subpopulations, including older patients, patients with suboptimal surgical resection, and patients with relapsed disease. 

The investigational treatment was well tolerated, and most patients did not experience serious side effects from the immunotherapy vaccine. Only 5 serious adverse events possibly related to the vaccine were reported: 3 cases of grade 2/3 intracranial edema, 1 case of grade 3 nausea, and 1 case of grade 3 infection [38].

## 4. Discussion

At first glance, the results of this study may seem surprising: in the last 18 years, it has been one of the first, if not the first, phase III studies to show significant increases in long-term survival for both newly diagnosed and relapsed GB, with an even greater benefit in the relapsed population. Specific poor prognosis subpopulations in this study showed unexpected benefits, including older patients and patients with significant residual disease where radical surgery was not possible.

However, some reflections regarding the methodology are required [54,55].

When considering PFS data, the study is negative, and the trial did not reach its prospectively defined primary endpoint. Therefore, from a purely formal point of view, the study should be declared negative.

The comparison of the investigational arm with an external control group should be considered a post-hoc retrospective analysis, suitable for generating hypotheses but not providing high-quality evidence. Non-randomized externally controlled studies are gradually becoming attractive because they are faster, cheaper, and limit the number of patients exposed to substandard or ineffective interventions, but they are inadequate for a phase III trial and require a pre-specified detailed protocol and robust statistical methods to minimize the risk of bias [56,57]. In this trial, the comparison of the active treatment arm with the external control population was not based on individual datasets from the selected randomized clinical trials; however, an indirect analysis was performed at the trial level with survival data reconstructed by an algorithm. The lack of individual patient data analysis represents a limitation: this trial does not provide a comparison on patient-level data, which compromises the quality of the evidence and the reliability of the results.

Furthermore, the artificial generation of the external control group resulted in impressive differences in the control population from the vaccine arm. This is a further major methodological limitation: the validity of external controls was compromised by the demographic characteristics of the comparison studies. The studies selected as an external control group had different patient characteristics, and this represents an important confounding factor.

Randomized controlled trials, even if difficult to conduct, are the gold standard for producing high-quality scientific evidence in phase III clinical trials and should always be pursued. The validity of external controls depends on the availability of high-quality patient-level data, methodological accuracy, and validation analyses to reduce the risk of distortions [58]. 

The DCVax-L trial included only patients who received gross or near total resection of the tumor mass, patients with disease confined to one hemisphere, and patients who had been off glucocorticoids for at least three weeks. All these criteria inevitably represent factors capable of favorably impacting survival; however, these inclusion criteria were not present in the studies used for comparison.

Moreover, patients with disease progression after completion of radiotherapy (which presumably have a poorer prognosis) were excluded from randomization in the DCVax-L trial, but this criterion was not included in all the comparison trials of the external control group; therefore, the vaccine trial selected patients with a more favorable prognosis, which could justify the long mOS observed. Similarly, other important patient characteristics, known as established prognostic factors, such as age, steroid use, performance status, and extent of resection, were not easily comparable between the two groups or were even missing in almost all selected trials. In several studies, the evaluation of MGMT methylation status was absent, and isocitrate dehydrogenase (IDH) mutational status has never been analyzed in any study.

The long randomization period implies that the criteria adopted for recruiting GB patients do not consider the 2016/2021 WHO classification. Consequently, the patients were not molecularly stratified, and this also creates a bias. In fact, it cannot be excluded that long-survivors might have had less aggressive tumors, for example, if IDH 1/2 mutated.

Certainly, we can agree with the investigators that PFS is not an adequate endpoint for immunotherapy studies because of the phenomenon of pseudoprogression, which is observed in approximately 40% of cases in vaccination trials [59]. Moreover, pseudoprogression is a frequent problem, especially in newly diagnosed MGMT-methylated GB; thus, PFS is a suboptimal endpoint for phase III trials, especially in this malignancy.

Despite the many doubts and perplexities that the methodology of this study raises, some cornerstones of the worth of its results remain unchanged: in a disease that is basically orphaned of treatments, such as GB, a therapy that might increase survival at the cost of low toxicities should not only arouse skepticism but also efforts and commitment to verify its efficacy with further appropriate clinical trials. Considering the extremely personalized nature of this treatment, when a GB patient treated with DCVax-L undergoes disease recurrence, a new, more specific vaccine batch can be prepared in order to restore disease control and effectively counteract any resistant clones.

The significant percentage of long-term survivors that is reported in this study could be consistent with an effect on immune memory by T lymphocytes; therefore, this new type of immunotherapy deserves further investigation. 

Dendritic cell vaccines are well suited to be used in combination therapeutic regimens, for example, in association with immune check-point blockade, oncolytic viruses, CAR-T therapy, Optune, other vaccines, etc. This is essential because all the latest studies conducted in neuro-oncology show that the most effective strategies to fight GB seem to be combination treatments and not monotherapies.

## 5. Conclusions

This innovative vaccine is undoubtedly promising; therefore, the methodological issues of the DCVax-L trial deserve our attention and, possibly, another confirmation trial.

Several changes have been made over the years (replacement of a randomized design with a synthetic control arm, removal of PFS as the primary endpoint, addition of a new study population, conduct of unplanned analyses), raising many questions about data interpretability [60].

Although we understand that the development of personalized vaccines is a complex and expensive process, the limitations of this trial reduce the reliability of the results and prevent drawing firm conclusions about the efficacy of the dendritic cell vaccine. 

To confirm the results of DCVax-L, it would be ideal to design two new trials, one in the newly diagnosed GB setting and another in the relapsed-disease setting, both randomized, for comparison with the standard of care. For example, it could be proposed to conduct a new randomized trial in the setting of recurrent GB, comparing the standard of care (lomustine) with the combination of DCVax-L plus an immune checkpoint inhibitor (PD-1/PD-L1 blocker or CTLA-4 inhibitor) as a strategy for boosting anti-tumor immune responses [28]. As expected in phase III trials, the primary endpoint of the study should be OS; secondary endpoints of the study should be PFS, quality of life, and patients’ reported outcomes.

We understand that randomized controlled trials strictly follow a pre-established protocol and risk being an overly rigid model because they fail to incorporate information that gradually becomes available. To overcome these rigidities, at least in part, adaptive trial designs can be explored, but randomized trials remain the gold standard for finding optimal answers to clinical questions. In considering the possibility of starting new trials; however, we should not overlook the great difficulties that dendritic cell vaccine entails: manufacturing is very expensive, requires adequate infrastructure, and involves an enormous expenditure of time, as demonstrated by the very long time required to conclude enrollment in the DCVax-L trial. Even in a hypothetical drug’s marketing phase, the problem of costs could be fundamental and demand a discussion between health authorities and pharmaceutical companies to ensure accessibility of the product to patients. 

## Data Availability

Data is unavailable.
